# Association between Body Composition and the Risk of Portopulmonary Hypertension Assessed by Computed Tomography in Patients with Liver Cirrhosis

**DOI:** 10.3390/jcm12103351

**Published:** 2023-05-09

**Authors:** Takao Miwa, Tatsunori Hanai, Kayoko Nishimura, Satoko Tajirika, Yuki Nakahata, Kenji Imai, Atsushi Suetsugu, Koji Takai, Mayumi Yamamoto, Masahito Shimizu

**Affiliations:** 1 Department of Gastroenterology/Internal Medicine, Graduate School of Medicine, Gifu University, Gifu 501-1194, Japan; 2 Health Administration Center, Gifu University, Gifu 501-1193, Japan; 3 Center for Nutrition Support & Infection Control, Gifu University Hospital, Gifu 501-1194, Japan; 4 Department of Gastroenterology, Asahi University Hospital, Gifu 501-1194, Japan; 5 Division for Regional Cancer Control, Graduate School of Medicine, Gifu University, Gifu 501-1194, Japan; 6 United Graduate School of Drug Discovery and Medical Information Sciences, Gifu University, Gifu 501-1194, Japan

**Keywords:** adipose tissue, muscle, obesity, pulmonary hypertension, sarcopenia, sarcopenic obesity

## Abstract

The aim of this study is to investigate the impact of body composition on the risk of portopulmonary hypertension using computed tomography (CT) in patients with liver cirrhosis. We retrospectively included 148 patients with cirrhosis treated at our hospital between March 2012 and December 2020. POPH high-risk was defined as main pulmonary artery diameter (mPA-D) ≥ 29 mm or mPA-D to ascending aorta diameter ratio ≥ 1.0, based on chest CT. Body composition was assessed using CT images of the third lumbar vertebra. The factors associated with POPH high-risk were evaluated using logistic regression and decision tree analyses, respectively. Among the 148 patients, 50% were females, and 31% were found to be high-risk cases on evaluation of chest CT images. Patients with a body mass index (BMI) of ≥25 mg/m^2^ had a significantly higher prevalence of POPH high-risk than those with a BMI < 25 mg/m^2^ (47% vs. 25%, *p* = 0.019). After adjusting for confounding factors, BMI (odds ratio [OR], 1.21; 95% confidence interval [CI], 1.10–1.33), subcutaneous adipose tissue index (OR, 1.02; 95% CI, 1.01–1.03), and visceral adipose tissue index (OR, 1.03; 95% CI, 1.01–1.04) were associated with POPH high-risk, respectively. In the decision tree analysis, the strongest classifier of POPH high-risk was BMI, followed by the skeletal muscle index. Body composition may affect the risk of POPH based on chest CT assessment in patients with cirrhosis. Since the present study lacked data on right heart catheterization, further studies are required to confirm the results of our study.

## 1. Introduction

Pulmonary hypertension (PH) is characterized by increased mean pulmonary artery pressure and can complicate the majority of cardiovascular and pulmonary disorders [1]. PH associated with portal hypertension is referred to as portopulmonary hypertension (POPH) and usually affects approximately 5% of patients with portal hypertension [1,2]. The precise pathogenesis of POPH is unclear; however, the major etiology of POPH is attributed to pulmonary vasoconstriction, which leads to increased mean pulmonary artery pressure and pulmonary vascular resistance [3]. Since POPH can cause right heart failure, the one and five-year survival rates of the patients without treatment are reported to be approximately 40% and 14%, respectively [4,5]. Therefore, the development of appropriate diagnostic and therapeutic strategies for POPH in the treatment of patients with cirrhosis is indispensable.

Drug therapy for POPH is equivalent to that for PH. For instance, macitentan, an endothelin receptor antagonist used to treat PH, has been reported to relieve pulmonary vascular resistance in patients with POPH [6]. However, the efficacy of PH-targeting agents for advanced POPH is limited. Liver transplantation (LT) may improve the prognosis of patients with POPH in the early phase; however, in severe POPH, LT is associated with postoperative complications and mortality [4,7]. A recent report has shown that PH-targeting agents improve cardiopulmonary hemodynamics, and a combination of these agents and LT may lead to favorable outcomes [8]. Therefore, the International Liver Transplant Society and the American Association for the Study of Liver Disease recommend screening for POPH for all LT candidates and treatment for severe POPH [4,7].

However, the screening and diagnosis of POPH are challenging owing to its low prevalence and nonspecific clinical manifestations, which could be misdiagnosed as liver-related complications [9,10]. In addition, the diagnosis of POPH requires right-heart catheterization, which is highly invasive and not routinely performed [1]. Therefore, screening for POPH is a crucial step for prioritizing high-risk patients who warrant further investigation. A recent study has revealed that measurement of the diameter of the main pulmonary artery (mPA-D) or evaluation of the ratio of mPA-D to the diameter of the ascending aorta (aAO-D) using chest computed tomography (CT) is useful for screening patients suspected to have POPH [11]. In this study, all three patients diagnosed with POPH in their institute fulfilled the CT-based criteria for screening POPH [11]. Since patients with cirrhosis are frequently investigated with CT to assess liver-related complications, screening for POPH using CT may be clinically advantageous in these patients. However, performing chest CT in all patients with cirrhosis is impractical in terms of cost and radiology exposure, and the use of chest CT should be limited to patients at risk for POPH. In addition, although obesity has been reported to affect pulmonary circulation potentially [12], the relationship between body composition and POPH has not been thoroughly investigated.

In the present study, we investigated the characteristics of cirrhotic patients with a high risk of POPH using chest CT and explored its association with body composition. Furthermore, the association of body composition, sarcopenia, and obesity with mortality was evaluated.

## 2. Materials and Methods

### 2.1. Study Design

This is a retrospective study including 148 Japanese cirrhosis patients who were admitted to the gastroenterology ward of Gifu University Hospital (Gifu, Japan) between March 2012 and December 2020. These patients were followed up until the last visit, death, or 24 March 2022, whichever occurred first. An opt-out method was used to obtain informed consent from all the patients due to the retrospective nature of this study. The Institutional Review Board of the Gifu University Graduate School of Medicine approved the study protocol (approval number: 2022-061). This study was conducted in accordance with the 2013 Declaration of Helsinki.

The inclusion criteria were patients with liver cirrhosis of any etiology who were aged ≥20 years and had undergone a CT examination. The exclusion criteria included a history of liver transplantation; active malignancies, including hepatocellular carcinoma; and comorbidities that may induce PH, cardiovascular disease, heart failure, lung disease, connective tissue disease, sarcoidosis, and thyroid disease. The diagnosis of liver cirrhosis was based on clinical, biochemical, and radiological features. The clinical characteristics and laboratory variables, including Child–Pugh score, a model for end-stage liver disease (MELD), and albumin-bilirubin (ALBI) scores, were assessed at the time of admission [13,14,15]. The risk of POPH, body composition, ascites, and portosystemic shunt was assessed using CT within 3 months of admission.

### 2.2. Assessment of POPH Risk Using Chest CT

The assessment of POPH risk using CT was based on the guidelines of the European Society of Cardiology and European Respiratory Society and a previous report [1,11]. The widest mPA-D, widest aAO-D, and inferior vena cava (IVC) were measured using CT, and the ratios of mPA-D and aAO-D (mPA-D/aAO-D) were calculated. Patients with mPA-D dilatation (≥29 mm) or increased mPA-D/aAO-D (≥1.0) were defined as ‘POPH high-risk’, whereas the other patients were defined as ‘POPH low-risk’ [11].

### 2.3. Assessment of Body Composition and Sarcopenia

**Figure 1:**
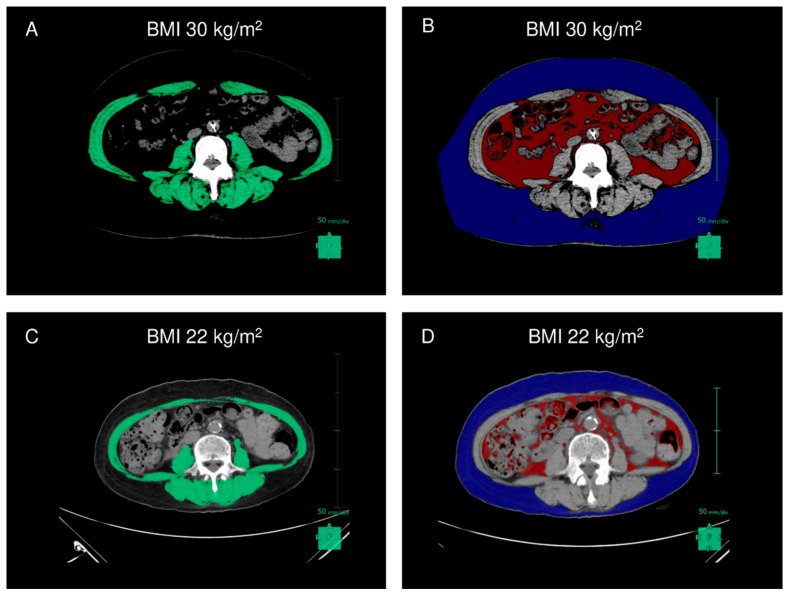
Assessment of skeletal muscle mass, subcutaneous adipose tissue, and visceral adipose tissue in patients with cirrhosis. The green, blue, and red areas represent SMI, SATI, and VATI in a female patient with cirrhosis (**A**,**B**) with and (**C**,**D**) without obesity. Abbreviations: BMI, body mass index; SATI, subcutaneous adipose tissue index; SMI, skeletal muscle index; VATI, visceral adipose tissue index.

Body composition was analyzed using CT images of the third lumbar vertebra and CT image analysis software (Synapse Vincent; Fujifilm, Tokyo, Japan). The skeletal muscle index (SMI), subcutaneous adipose tissue index (SATI), visceral adipose tissue index (VATI), and total adipose tissue index (TATI) were calculated from the skeletal muscle mass, subcutaneous adipose tissue, visceral adipose tissue, and total adipose tissue area at the third lumbar vertebra, respectively (Figure 1) [16,17,18]. Patients with both reduced SMI (<42 cm^2^/m^2^ in males and <38 cm^2^/m^2^ in females) and handgrip strength (<28 kg in males and <18 kg in females) were diagnosed with sarcopenia based on the criteria proposed by the Japan Society of Hepatology [19,20]. Patients with body mass index (BMI) ≥ 25 mg/m^2^ were diagnosed as obese.

### 2.4. Statistical Analyses

Baseline characteristics were expressed as medians and interquartile ranges for quantitative variables and as frequency and percentages for qualitative variables. Quantitative and qualitative variables were compared using the Mann–Whitney *U* and chi-square tests, respectively. The ability to identify POPH high-risk was evaluated using receiver operating characteristic (ROC) curve analysis, and the results were presented as the area under the ROC curve (AUC). The optimal cutoff values were assessed using the Youden index. Factors associated with POPH high-risk were analyzed using logistic regression analysis, and the results were expressed as odds ratios (ORs) with 95% confidence intervals (CIs). A decision-tree analysis was carried out to reveal the classifier associated with POPH high-risk. The Cox proportional hazards regression model was used for evaluating survival, and the results were expressed as hazard ratios (HRs) with 95% CIs. Survival curves were estimated using the Kaplan–Meier method, and the survival was compared using the log-rank test. Two-sided tests with a *p-*value < 0.05 were considered statistically significant. Statistical analyses were performed using JMP Pro version 16.2.0 (SAS Institute Inc., Cary, NC, USA) and R version 4.1.3 software (The R Foundation for Statistical Computing, Vienna, Austria).

## 3. Results

### 3.1. Baseline Characteristics of Patients

Among the 148 patients, 74 (50%) were females, and the median age and BMI were 70 years and 22.8 kg/m^2^, respectively. The major reasons for admission were treatment of varices (29%), ascites (22%), hepatic encephalopathy (6%), portosystemic shunt (5%), and other reasons (37%). Cirrhosis was attributed to hepatitis C virus (27%), alcohol-related liver disease (26%), hepatitis B virus (12%), nonalcoholic steatohepatitis (8%), autoimmune hepatitis or primary biliary cholangitis (7%), and other causes (22%). Ascites, hepatic encephalopathy, esophageal varices, and portosystemic shunts were present in 51%, 12%, 80%, and 11% of the patients, respectively. The median Child–Pugh, MELD, and ALBI scores were 7, 9, and −1.90, respectively. The median SMI, SATI, VATI, and TATI were 42 kg/m^2^, 35 kg/m^2^, 34 kg/m^2^, and 71 kg/m^2^, respectively. Sarcopenia was observed in 28% of patients. Chest CT evaluation revealed that the median mPA-D, aAO-D, IVC, and mPA-D/aAO-D were 33, 27, 27 mm, and 0.80, respectively (Table 1).

### 3.2. Comparison of Cirrhotic Patients with Low-Risk and High-Risk for POPH Based on Chest CT

A comparison of the characteristics of patients with POPH low- and high-risk assessed using chest CT is shown in Table 1. Regarding the POPH risk assessment, the prevalence of patients with mPA-D dilatation and increased mPA-D/aAo-D was 29% (*n* = 43) and 7% (*n* = 11), respectively, and 31% of patients (*n* = 46) had at least one of these. Most POPH high-risk patients were females with higher BMI, higher prevalence of portosystemic shunt, dilated IVC, and lower MELD scores than low-risk patients. Regarding body composition, POPH high-risk patients had significantly higher SATI, VATI, and TATI values than low-risk patients, whereas there was no significant difference in SMI and sarcopenia between these groups.

### 3.3. Comparison of the Characteristics of Cirrhotic Patients with and without Obesity

**Figure 2:**
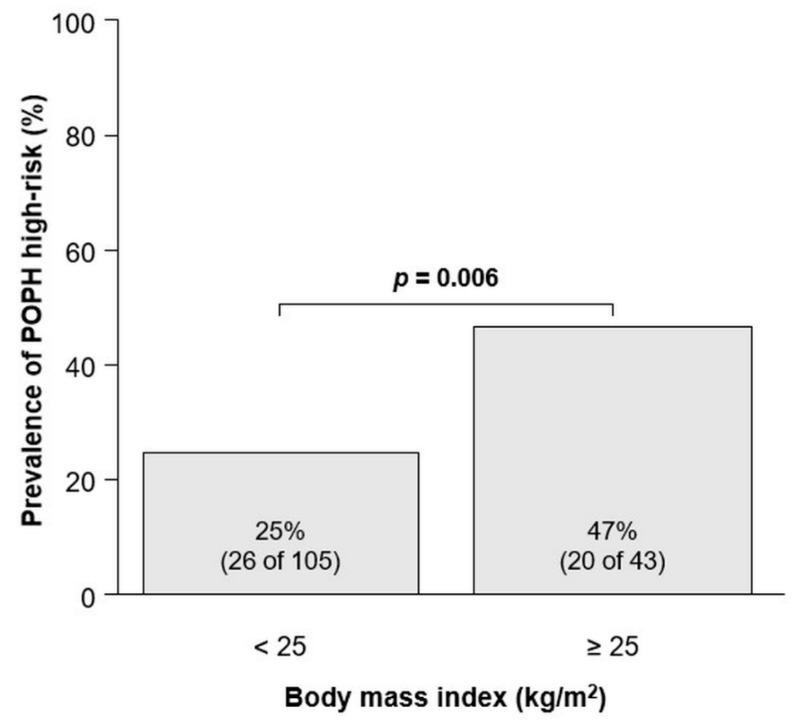
Prevalence of POPH high-risk divided by BMI. The groups were compared using the Mann–Whitney *U* test. Abbreviations: BMI, body mass index; POPH, portopulmonary hypertension.

Since higher BMI and increased levels of adipose tissue were found to be associated with POPH high-risk (Table 1), the next study examined the impact of obesity on patient background, including risk factors for POPH. As listed in Table 2, patients with obesity were more females and had a higher prevalence of hypertension and portosystemic shunt but a lower prevalence of ascites than those without obesity. In addition, SMI, SATI, VATI, and TATI were significantly higher in patients with obesity than in those without obesity. Regarding chest CT findings, the mPA-D (29 vs. 27 mm, *p* = 0.011) and IVC (29 vs. 27 mm, *p* = 0.019) were significantly dilated in obese patients than those without obesity. Consequently, the prevalence of POPH high-risk was significantly higher in obese patients than in non-obese patients (47% vs. 25%, *p* = 0.009; Figure 2).

### 3.4. Impact of Body Composition on POPH High-Risk in Cirrhotic Patients

Multivariate analysis showed that BMI (HR, 1.16; 95% CI, 1.05–1.28; *p* = 0.004) was independently associated with POPH risk after adjusting for sex, portosystemic shunt, and IVC. Similar results were also found for SATI (OR, 1.02; 95% CI, 1.01–1.03; *p* = 0.010), VATI (OR, 1.02; 95% CI, 1.01–1.23; *p* = 0.006), and TATI (OR, 1.11; 95% CI, 1.00–1.02; *p* = 0.003), whereas no such association was found for SMI (Table 3). Details of the univariate and multivariate analyses are shown in Tables S1 and S2.

**Figure 3:**
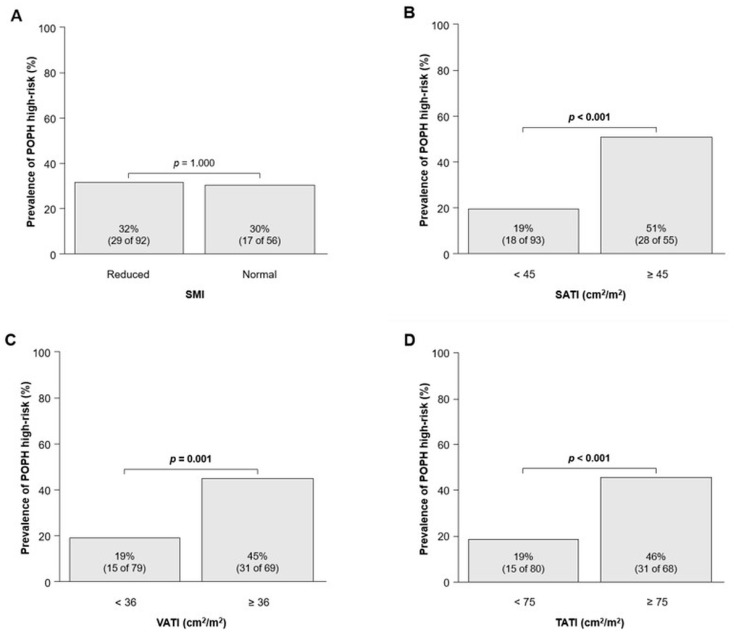
Prevalence of POPH high-risk divided by (**A**) SMI, (**B**) SATI, (**C**) VATI, and (**D**) TATI. The groups were compared using the Mann–Whitney *U* test. Abbreviations: SATI, subcutaneous adipose tissue index; SMI, skeletal muscle index; TATI, total adipose tissue index; VATI, visceral adipose tissue index.

The AUCs of SATI, VATI, and TATI to identify POPH high-risk were 0.69 (95% CI, 0.60–0.79), 0.69 (95% CI, 0.60–0.79), and 0.72 (95% CI, 0.63–0.81), respectively. The optimal cutoff values of SATI, VATI, and TATI for identifying POPH high-risk were 45 kg/m^2^, 36 kg/m^2^, and 75 kg/m^2^, respectively. There was no association between SMI and the incidence of POPH high-risk (Figure 3A); however, patients with or above the cutoff values of SATI, VATI, and TATI had a higher incidence of POPH high-risk than those below the cutoff values (Figure 3B–D).

### 3.5. Decision Tree Analysis for Factors Associated with POPH High-Risk

**Figure 4:**
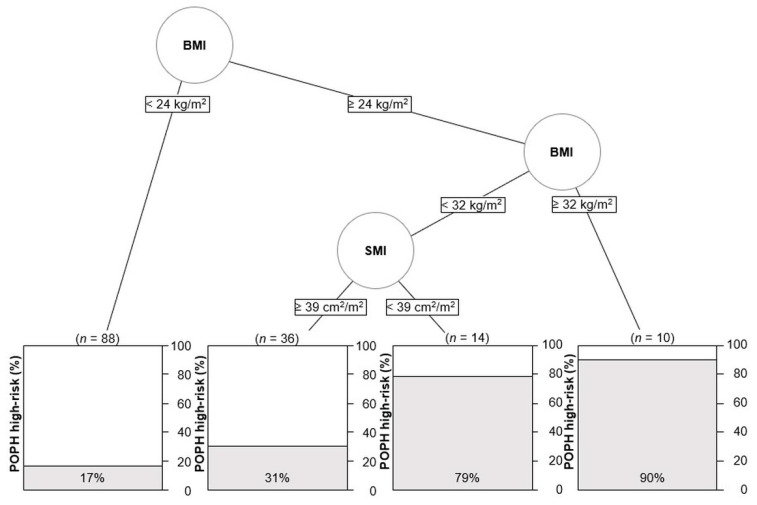
A decision tree analysis to identify patients with POPH high-risk. Abbreviations; BMI, body mass index; POPH, portopulmonary hypertension; SMI, skeletal muscle index.

Next, decision tree analysis was performed to examine the factors preferentially associated with POPH high-risk. Based on the analysis, BMI was selected as the most important classifier to identify patients with high POPH risk. Patients with a BMI ≥ 32 kg/m^2^ had the highest prevalence of POPH high-risk (90%), whereas patients with a BMI < 24 kg/m^2^ had the lowest prevalence (17%). Among the patients with BMI ≥ 24 kg/m^2^ and less than 32 kg/m^2^, SMI ≥ 39 cm^2^/m^2^ was identified as an important classifier for POPH high-risk (Figure 4).

### 3.6. Association between Body Composition and Survival in Patients with Cirrhosis

The Cox proportional hazards regression model was used to evaluate the association between body composition and survival in patients with cirrhosis (Table S3). The analyses revealed that although SATI (HR, 0.98; 95% CI, 0.96–0.99; *p* = 0.004) was associated with survival in patients with cirrhosis, no such association was found for SMI and VATI.

### 3.7. Association between Sarcopenia, Obesity, and Survival in Patients with Cirrhosis

To assess the association between sarcopenia, obesity, and survival, the patients were divided into four groups as follows: No sarcopenia/No obesity group (*n* = 72); No sarcopenia/Obesity group (*n* = 35); Sarcopenia/No obesity group (*n* = 33); and Sarcopenia/Obesity group (*n* = 8). As for survival, body composition had a significant association with survival among these groups (*p* < 0.001; Figure S1). The Sarcopenia/No obesity group had significantly worse survival than the No sarcopenia/No obesity group (HR, 4.06; 95% CI, 1.94–8.47; *p* < 0.001). A similar tendency was shown in the Sarcopenia/Obesity group (HR, 3.94; 95% CI, 0.87–10.66; *p* = 0.083); however, no such association was found in the No sarcopenia/Obesity group (HR, 1.24; 95% CI, 0.50–3.07; *p* = 0.646).

## 4. Discussion

Since the mortality of candidates for LT with POPH is extremely high, there have been many studies on POPH among this population [7]. In addition, recent studies have attempted to explore POPH in patients with more preserved liver functional reserves [9,10]. For early diagnosis and intervention of POPH, it is necessary to establish a simple screening method to identify patients with POPH [21]. A recent study reported a risk assessment for POPH using chest CT scans, which are frequently performed in patients with cirrhosis [11]. Moreover, CT is an accurate and established method to evaluate body composition, including muscle mass and fat mass, in patients with cirrhosis [16,17]. Body composition is known to be involved in the pathophysiology of patients with cirrhosis [16,17]; however, studies on its relationship with POPH have been scarce. Therefore, we examined the association between body composition and the risk of POPH, defined by mPA-D dilatation and increased mPA-D/aAo-D [11], in cirrhotic patients using CT images.

The risk factors of POPH in patients with cirrhosis remain unclear. Previous studies have reported that female sex, etiology of cirrhosis, history of splenectomy, and portosystemic shunt may be associated with POPH [22,23,24], but it is uncertain whether liver functional reserves, the severity of liver fibrosis, and abnormalities in body composition are risk factors for POPH [9,10]. The results of the present study were the first evidence to show that cirrhotic patients with obesity had higher mPA-D and mPA-D/aAO-D, both of which are associated with the risk of POPH [11]. Detailed body composition analysis, adjusted for previously reported risk factors such as female sex, portosystemic shunt, and IVC dilation [2,11,24], also showed an increased risk of POPH in cirrhotic patients with increased SATI, VATI, and TATI. To the best of our knowledge, no report has focused on the association between POPH and obesity or increased adipose tissue. However, it has been reported in a large American hospitalized cohort that BMI strongly affects pulmonary vascular hemodynamics [12]. Studies have demonstrated that inflammation and endothelin-1 play an important role in the pathophysiology of POPH [25,26,27]. Since obesity is known to induce systemic inflammation and increase vascular endothelin-1 and endothelin-1 receptor expression [28], adipose tissue can function as a modulator of PH. This evidence supports our new findings that obesity and increased adipose tissue may be associated with the risk of POPH in patients with cirrhosis.

Another intriguing finding of the present study is that reduced skeletal muscle mass and obesity may be important classifiers for identifying patients at risk of POPH. Sarcopenia, characterized by reduced muscle mass and strength, has a significant role in the development of complications and outcomes in patients with cirrhosis [16]. However, to date, no studies have confirmed the association between sarcopenia and POPH. In meta-analyses, approximately 15.5% to 34% of patients with chronic obstructive pulmonary disease have sarcopenia, and these patients with sarcopenia have lower expiratory volume and poorer exercise tolerance [29]. Another study reported that lung transplant candidates with sarcopenia had an increased risk of delisting or mortality [30]. This evidence suggests that pulmonary disease’s severity negatively impacts skeletal muscle. Furthermore, sarcopenia itself may strongly impair the prognosis of patients with pulmonary disease. In fact, patients with sarcopenia and without obesity showed worse survival than those without them in our study. Since POPH can lead to right heart failure [3], patients with POPH may easily develop sarcopenia resulting from exercise disability. Our findings provide the first evidence that reduced muscle mass may stratify the risk of POPH in patients with cirrhosis.

Other interesting findings in the present study are that female sex, portosystemic shunt, and IVC diameter were also associated with POPH risk using CT images. A prospective study has shown that the risk of POPH in females is approximately three times higher than that in males [22]. Similarly, our results showed that the probability of POPH high-risk in females is approximately two times higher than that in males. Another retrospective study has revealed that large portosystemic shunt is associated with the severity of POPH and response to treatment of POPH [24]. Furthermore, the study, which suggested the use of CT images for POPH screening, has also demonstrated that female sex, portosystemic shunt, and IVC diameter are independently associated with POPH risk in the multivariate logistic regression model [11]. Therefore, the results of the present study not only provided new evidence in body composition and POPH risk but also strengthened the evidence of previously reported pathophysiology of POPH.

As for the association between body composition and survival, we additionally found that SATI was associated with survival in patients with cirrhosis. This is supported by a recent study that revealed that there is a U-shaped association between SATI and mortality in patients with cirrhosis [31]. Furthermore, this study also revealed the prognostic significance of sarcopenic obesity in patients with cirrhosis. Given their robust impact on survival, assessing body composition, sarcopenia, and obesity is a fundamental step in stratifying the risk of adverse outcomes in patients with cirrhosis.

The limitations of our study are as follows. Firstly, this single-center retrospective study may include bias and confounding factors. Secondly, since we did not perform the right heart catheterization in this study, the association between body composition and pulmonary vascular hemodynamics was unsatisfactory in patients with cirrhosis. A previous study has suggested the usefulness of chest CT findings for assessing POPH risk [11]. However, the assessment of POPH risk using CT findings should be considered with caution because we found that chest CT findings of PH are affected by body composition. Furthermore, since the latest guideline recommends the use of echocardiography for the screening of POPH [32], the usefulness of echocardiography for screening POPH and its association with body composition should be evaluated in future studies. Third, body composition has variations, and our findings may not be adapted to other ethnicities and legions. Therefore, further multicenter studies with right heart catheterization are required to validate the relationship between body composition and POPH in patients with cirrhosis.

## 5. Conclusions

In conclusion, obesity and reduced skeletal muscle mass may strongly affect the expansion of mPA-D assessed using chest CT. Abnormal body composition is closely associated with prognosis and complications in patients with cirrhosis. Therefore, assessment of body composition is extremely important in diagnosing cirrhosis complications and in practicing prevention and treatment to improve the prognosis of cirrhosis patients. Since the present study lacked data on right heart catheterization, further studies are required to confirm the results of our study.

## Figures and Tables

**Table 1:** Baseline characteristics of patients with cirrhosis categorized based on POPH risk.

Characteristic	All Patients	POPH Low-Risk	POPH High-Risk	*p-*Value
	(*n* = 148)	(*n* = 102)	(*n* = 46)	
Age (years)	70 (58–76)	70 (59–76)	65 (65–78)	0.545
Female, *n* (%)	74 (50)	44 (43)	30 (65)	0.013
Body mass index (kg/m^2^)	22.8 (21.7–25.5)	22.3 (21.4–24.5)	24.5 (22.3–28.2)	<0.001
Etiology of cirrhosis, *n* (%)				0.344
HCV	40 (27)	23 (23)	17 (37)	
HBV	18 (12)	13 (13)	5 (11)	
ALD	38 (26)	31 (30)	7 (15)	
AIH or PBC	11 (7)	8 (8)	3 (7)	
NASH	12 (8)	8 (8)	4 (9)	
Others	29 (20)	19 (19)	10 (22)	
Type 2 diabetes mellitus, *n* (%)	43 (29)	32 (31)	11 (24)	0.436
Hypertension, *n* (%)	42 (28)	26 (25)	16 (35)	0.246
Dyslipidemia, *n* (%)	7 (5)	6 (6)	1 (2)	0.325
Ascites, *n* (%)	75 (51)	54 (53)	21 (46)	0.479
Hepatic encephalopathy, *n* (%)	18 (12)	15 (15)	3 (7)	0.158
Esophageal varices, *n* (%)	119 (80)	84 (82)	35 (76)	0.379
Portosystemic shunt, *n* (%)	17 (11)	8 (8)	9 (20)	0.039
aAO-D (mm)	33 (31–36)	33 (31–36)	34 (32–36)	0.410
mPA-D (mm)	27 (25–29)	26 (24–27)	30 (29–32)	<0.001
mPA-D/aAO-D	0.80 (0.73–0.90)	0.75 (0.71–0.85)	0.92 (0.85–0.99)	<0.001
IVC (mm)	27 (25–30)	27 (25–30)	29 (26–31)	0.008
Child–Pugh score	7 (5–9)	7 (5–10)	7 (5–9)	0.413
Child–Pugh class (A/B/C)	63/51/34	43/33/26	20/18/8	0.512
Laboratory test				
MELDscore	9 (7–12)	10 (7–13)	8 (7–11)	0.030
ALBIscore	−1.90 (−2.38–−1.18)	−1.88 (−2.37–−1.20)	−1.95 (−2.43–−1.08)	0.590
Internationalnormalizedratio	1.12 (1.02–1.26)	1.15 (1.02–1.30)	1.08 (1.01–1.24)	0.154
Platelet (10^9^/L)	94 (62–143)	93 (63–140)	104 (54–155)	0.868
Creatinine (mg/dL)	0.74 (0.60–0.95)	0.74 (0.60–0.98)	0.72 (0.59–0.88)	0.463
Albumin (g/dL)	3.2 (3.5–3.8)	3.2 (2.5–3.8)	3.2 (2.4–3.8)	0.581
Bilirubin (mg/dL)	1.2 (0.7–1.7)	1.2 (0.9–1.8)	1.0 (0.8–1.5)	0.194
Sodium (meq/L)	139 (137–140)	138 (137–140)	139 (136–141)	0.080
Ammonia (μg/dL)	58 (41–93)	56 (41–87)	66 (42–98)	0.397
HemoglobinA1c (%)	5.5 (5.0–6.2)	5.0 (5.6–5.5)	5.5 (5.0–6.2)	0.493
Triglycerides (mg/dL)	51 (67–104)	68 (52–98)	64 (47–106)	0.714
Totalcholesterol (mg/dL)	140 (114–169)	143 (114–169)	138 (120–170)	0.838
Systolic blood pressure (mmHg)	118 (109–131)	118 (109–133)	119 (108–130)	0.547
Diastolic blood pressure (mmHg)	68 (59–77)	68 (59–78)	68 (58–77)	0.553
SMI (cm^2^/m^2^)	42 (37–48)	42 (36–47)	42 (37–50)	0.493
SATI (cm^2^/m^2^)	35 (22–63)	32 (20–50)	53 (30–85)	<0.001
VATI (cm^2^/m^2^)	34 (20–56)	31 (17–50)	45 (30–69)	<0.001
TATI (cm^2^/m^2^)	71 (45–124)	65 (38–103)	103 (68–151)	<0.001
Handgrip strength (kg)	21 (16–29)	21 (16–29)	21 (16–27)	0.688
Sarcopenia, *n* (%)	41 (28)	30 (29)	11 (24)	0.489

Values are presented as frequency (percentage) or medians (interquartile ranges). Abbreviations: aAO-D, ascending aorta diameter; AIH, autoimmune hepatitis; ALBI, albumin-bilirubin; ALD, alcohol-related liver disease; HBV, hepatitis B virus; HCV, hepatitis C virus; IVC, inferior vena cava; MELD, model for end-stage liver disease; mPA-D, main pulmonary artery diameter; NASH, nonalcoholic steatohepatitis; PBC, primary biliary cholangitis; POPH, portopulmonary hypertension; SATI, subcutaneous adipose tissue index; SMI, skeletal muscle index; TATI, total adipose tissue index; VATI, visceral adipose tissue index.

**Table 2:** Baseline characteristics of patients with cirrhosis categorized based on obesity status.

Characteristic	No Obesity	Obesity	*p-*Value
	(*n* = 105)	(*n* = 43)	
Age (years)	70 (59–76)	66 (54–76)	0.637
Female, *n* (%)	47 (45)	27 (63)	0.046
Body mass index (kg/m^2^)	22.0 (21.0–23.4)	27.0 (25.7–31.5)	<0.001
Etiology of cirrhosis, *n* (%)			0.018
HCV	23 (24)	15 (35)	
HBV	15 (14)	3 (7)	
ALD	31 (30)	7 (16)	
AIH or PBC	9 (9)	2 (5)	
NASH	4 (4)	8 (19)	
Others	21 (20)	8 (19)	
Type 2 diabetes mellitus, *n* (%)	28 (27)	15 (35)	0.318
Hypertension, *n* (%)	24 (23)	18 (42)	0.019
Dyslipidemia, *n* (%)	4 (4)	3 (7)	0.410
Ascites, *n* (%)	62 (59)	13 (30)	0.002
Esophageal varices, *n* (%)	82 (78)	37 (86)	0.269
Portosystemic shunt, *n* (%)	7 (7)	10 (23)	0.004
aAO-D (mm)	33 (31–36)	33 (32–36)	0.333
mPA-D (mm)	27 (24–29)	29 (25–30)	0.011
mPA-D/aAO-D	0.80 (0.72–0.89)	0.84 (0.74–0.94)	0.198
POPH high-risk, *n* (%)	26 (25)	20 (47)	0.009
IVC (mm)	27 (25–30)	29 (26–31)	0.019
Child–Pugh score	7 (5–10)	6 (5–8)	0.156
Child–Pugh class (A/B/C)	40/39/26	2020/12/8	0.228
Laboratory test			
MELDscore	9 (7–13)	9 (7–12)	0.769
ALBIscore	−1.85 (−2.39–−1.16)	−2.06 (−2.36–−1.17)	0.553
Internationalnormalizedratio	1.12 (1.01–1.26)	1.12 (1.05–1.30)	0.615
Platelet (10^9^/L)	94 (62–153)	91 (62–129)	0.661
Creatinine (mg/dL)	0.75 (0.62–0.98)	0.67 (0.51–0.86)	0.046
Albumin (g/dL)	3.2 (2.5–3.8)	3.2 (2.4–3.8)	0.479
Bilirubin (mg/dL)	1.1 (0.8–1.7)	1.3 (0.8–1.6)	0.703
Sodium (meq/L)	138 (136–140)	139 (137–141)	0.026
Ammonia (μg/dL)	57 (39–87)	66 (48–101)	0.080
HemoglobinA1c (%)	5.5 (4.9–6.2)	5.8 (5.1–6.5)	0.097
Triglycerides (mg/dL)	65 (51–98)	71 (49–130)	0.594
Totalcholesterol (mg/dL)	140 (112–171)	142 (120–166)	0.724
Systolic blood pressure (mmHg)	117 (107–129)	127 (112–135)	0.026
Diastolic blood pressure (mmHg)	67 (59–77)	72 (59–80)	0.401
SMI (cm^2^/m^2^)	40 (36–45)	48 (41–52)	<0.001
SATI (cm^2^/m^2^)	30 (19–41)	75 (47–115)	<0.001
VATI (cm^2^/m^2^)	30 (18–77)	58 (40–77)	<0.001
TATI (cm^2^/m^2^)	63 (41–89)	138 (103–166)	<0.001
Handgrip strength (kg)	21 (17–29)	22 (15–28)	0.571
Sarcopenia, *n* (%)	33 (31)	8 (19)	0.104

Values are presented as frequency (percentage) or medians (interquartile ranges). Abbreviations: aAO-D, ascending aorta diameter; AIH, autoimmune hepatitis; ALBI, albumin-bilirubin; ALD, alcohol-related liver disease; HBV, hepatitis B virus; HCV, hepatitis C virus; IVC, inferior vena cava; MELD, model for end-stage liver disease; mPA-D, main pulmonary artery diameter; NASH, nonalcoholic steatohepatitis; PBC, primary biliary cholangitis; POPH, portopulmonary hypertension; SATI, subcutaneous adipose tissue index; SMI, skeletal muscle index; TATI, total adipose tissue index; VATI, visceral adipose tissue index.

**Table 3:** Impact of body composition on POPH high-risk in patients with cirrhosis.

Characteristic	Univariate		Multivariate	
	OR (95% CI)	*p-*Value	OR (95% CI)	*p-*Value ^a^
Body mass index (kg/m^2^)	1.21 (1.10–1.33)	<0.001	1.16 (1.05–1.28)	0.004
SATI (cm^2^/m^2^)	1.02 (1.01–1.03)	<0.001	1.02 (1.00–1.03)	0.010
VATI (cm^2^/m^2^)	1.03 (1.01–1.04)	<0.001	1.02 (1.01–1.23)	0.006
TATI (cm^2^/m^2^)	1.02 (1.01–1.02)	<0.001	1.11 (1.00–1.02)	0.003
SMI (cm^2^/m^2^)	1.01 (0.98–1.05)	0.439		

^a^ Adjusted for sex, portosystemic shunt, and inferior vena cava. Abbreviations: CI, confidence interval; OR, odds ratio; POPH, portopulmonary hypertension; SATI, subcutaneous adipose tissue index; SMI, skeletal muscle index; TATI, total adipose tissue index; VATI, visceral adipose tissue index.

## Data Availability

The datasets generated and/or analyzed during the current study are available from the corresponding author upon reasonable request.

## Supplementary Materials

The following supporting information can be downloaded at: https://www.mdpi.com/article/10.3390/jcm12103351/s1, Table S1: Univariate analysis predicting POPH high-risk in patients with cirrhosis; Table S2: Multivariate analysis for predicting POPH high-risk in patients with cirrhosis; Table S3: Impact of body composition on survival in patients with cirrhosis; Figure S1: Association between sarcopenia, obesity, and survival in patients with cirrhosis.
