# Supplementary material for: Association between Body Composition and the Risk of Portopulmonary Hypertension Assessed by Computed Tomography in Patients with Liver Cirrhosis
Source: J Clin Med. 2023 May 9;12(10):3351. doi: 10.3390/jcm12103351 (PMC10219301; doi:10.3390/jcm12103351)
Supplement: Supplementary file 1 [file jcm-12-03351-s001.zip › jcm-2292504-supplementary.pdf]

Supplementary Materials

Table S1. Univariate analysis predicting POPH high-risk in patients with cirrhosis.

Characteristic	OR (95% CI)	*p* Value
Age (years)	0.99 (0.96–1.02)	0.380
Female sex	2.47 (1.20–5.09)	0.014
Body mass index (kg/m^2)	1.21 (1.10–1.33)	<0.001
Etiology of cirrhosis		
HBV [a]	1.00	
HCV	1.92 (0.57–6.43)	0.289
ALD	0.59 (0.16–2.19)	0.428
AIH or PBC	0.98 (0.18–5.23)	0.976
NASH	1.30 (0.27–6.33)	0.745
Others	1.37 (0.38–4.94)	0.632
Type 2 diabetes mellitus	0.69 (0.31–1.52)	0.356
Hypertension, *n* (%)	1.56 (0.73–3.31)	0.248
Dyslipidemia, *n* (%)	0.36 (0.04–3.04)	0.345
Ascites, *n* (%)	0.75 (0.37–1.50)	0.412
Esophageal varices	0.68 (0.29–1.59)	0.376
Portosystemic shunt, *n* (%)	2.86 (1.02–7.97)	0.045
IVC (mm)	1.12 (1.03–1.22)	0.010
Child–Pugh score	0.93 (0.80–1.08)	0.353
Laboratory test		
MELD score	0.91 (0.81–1.01)	0.082
ALBI score	0.84 (0.54–1.31)	0.441
International normalized ratio	0.23 (0.03–1.91)	0.175
Platelet (10^9/L)	1.00 (1.00–1.00)	0.269
Creatinine (mg/dL)	0.71 (0.31–1.63)	0.417
Albumin (g/dL)	0.15 (0.75–1.77)	0.522
Bilirubin (mg/dL)	0.90 (0.67–1.21)	0.480
Sodium (meq/L)	1.06 (0.95–1.18)	0.299
Ammonia (μg/dL)	1.00 (1.00–1.01)	0.377
Hemoglobin A1c (%)	0.89 (0.57–1.35)	0.553
Triglycerides (mg/dL)	1.00 (1.00–1.01)	0.487
Total cholesterol (mg/dL)	1.00 (1.00–1.01)	0.644
Systolic blood pressure (mmHg)	0.99 (0.97–1.02)	0.526
Diastolic blood pressure (mmHg)	0.99 (0.96–1.02)	0.557
SMI (cm^2/m^2)	1.01 (0.98–1.05)	0.439
SATI (cm^2/m^2)	1.02 (1.01–1.03)	< 0.001
VATI (cm^2/m^2)	1.03 (1.01–1.04)	< 0.001
TATI (cm^2/m^2)	1.02 (1.01–1.02)	< 0.001
Handgrip strength (kg)	0.99 (0.95–1.03)	0.531
Sarcopenia, *n* (%)	0.75 (0.34–1.68)	0.490

[a] Reference group. Abbreviations: AIH, autoimmune hepatitis, ALBI, albumin-bilirubin; ALD, alcoholic liver disease; CI, confidence interval; HBV, hepatitis B virus; HCV, hepatitis C virus; IVC, inferior vena cava; MELD, model for end-stage liver disease; NASH, nonalcoholic steatohepatitis; OR, odds ratio; PBC, primary biliary cholangitis; POPH, portopulmonary hypertension, SATI, subcutaneous adipose tissue index; SMI, skeletal muscle index, TATI, total adipose tissue index; VATI, visceral adipose tissue index.

Table S2. Multivariate analysis for predicting POPH high-risk in patients with cirrhosis.

Characteristic	OR (95% CI)	*p* Value
Model 1		
Female sex	2.03 (0.91–4.53)	0.084
Portosystemic shunt	2.01 (0.65–6.23)	0.225
IVC (mm)	1.11 (1.01–1.22)	0.032
Body mass index (kg/m^2)	1.16 (1.05–1.28)	0.004
Model 2		
Female sex	1.67 (0.70–3.95)	0.247
Portosystemic shunt	1.78 (0.57–5.56)	0.319
IVC (mm)	1.12 (1.02–1.24)	0.023
SATI (cm^2/m^2)	1.02 (1.00–1.03)	0.010
Model 3		
Female sex	2.67 (1.21–5.86)	0.015
Portosystemic shunt	1.96 (0.64–5.97)	0.236
IVC (mm)	1.12 (1.01–1.23)	0.025
VATI (cm^2/m^2)	1.02 (1.01–1.04)	0.006
Model 4		
Female sex	1.89 (0.83–4.28)	0.129
Portosystemic shunt	1.72 (0.55–5.38)	0.354
IVC (mm)	1.11 (1.01–1.23)	0.029
TATI (cm^2/m^2)	1.11 (1.00–1.02)	0.003

Abbreviations: CI, confidence interval; IVC, inferior vena cava; OR, odds ratio; POPH, portopulmonary hypertension; SATI, subcutaneous adipose tissue index; TATI, total adipose tissue index; VATI, visceral adipose tissue index.

Table S3. Impact of body composition on survival in patients with cirrhosis.

Characteristic	HR (95% CI)	*p* Value
SMI (cm^2/m^2)	0.97 (0.94–1.01)	0.188
SATI (cm^2/m^2)	0.98 (0.96–0.99)	0.004
VATI (cm^2/m^2)	0.99 (0.97–1.00)	0.069
TATI (cm^2/m^2)	0.99 (0.98–1.00)	0.007

Abbreviations: CI, confidence interval; HR, hazard ratio; SATI, subcutaneous adipose tissue index; SMI, Skeletal muscle index; TATI, total adipose tissue index; VATI, visceral adipose tissue index.

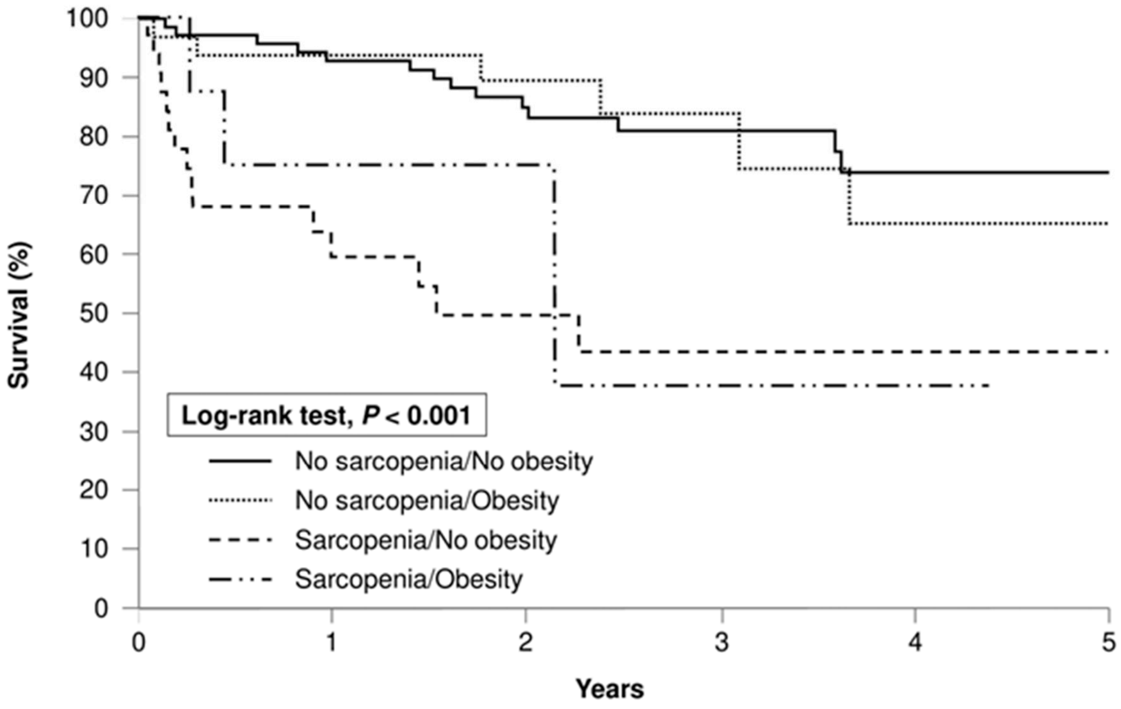

Figure S1. Association between sarcopenia, obesity, and survival in patients with cirrhosis. Survival between groups were compared using the log-rank test and survival curves were estimated using the Kaplan–Meier method.
